# *Sphingomonas degradans* sp. nov. and *Sphingomonas paludis* sp. nov., isolated from the Han River and a wetland in South Korea

**DOI:** 10.71150/jm.2510010

**Published:** 2026-01-31

**Authors:** Seung-Tae Kim, Miryung Kim, Chang-Jun Cha

**Affiliations:** Department of Systems Biotechnology and Center for Antibiotic Resistome, Chung-Ang University, Anseong 17546, Republic of Korea

**Keywords:** *Sphingomonas*, *Sphingomonas degradans*, *Sphingomonas paludis*, novel species

## Abstract

Two novel bacterial strains, designated CJ20^T^ and CJ99^T^, belonging to the genus *Sphingomonas*, were isolated from the Han River in South Korea and a wetland in South Korea, respectively. Cells of both strains were Gram-stain-negative, aerobic, non-motile and yellow-pigmented. Strains were shown to grow optimally at 30˚C and pH 7 in the absence of NaCl on tryptic soy medium. Phylogenetic analysis based on 16S rRNA gene sequences showed that strains CJ20^T^ and CJ99^T^ belonged to the genus *Sphingomonas* and were most closely related to *S. asaccharolytica* Y-345^T^ and *Sphingomonas koreensis* JSS26^T^ with 97.87% and 97.58% 16S rRNA gene sequence similarities, respectively. Average nucleotide identity and digital DNA-DNA hybridization values of strain CJ20^T^ with *S. asaccharolytica* Y-345^T^ were 74.1% and 15.9%, respectively and those values of strain CJ99^T^ with *S. koreensis* JSS26^T^ were 73.9% and 15.6%, respectively. Both strains contained ubiquinone (Q-10) as the predominant respiratory quinone. The major polar lipids of strains CJ20^T^ and CJ99^T^ comprised phosphatidylethanolamine, diphosphatidylglycerol, phosphatidylglycerol, and sphingoglycolipid. The predominant fatty acids of both strains were summed feature 8 (C_18:1_ *ω7c* and/or C_18:1_ *ω6c*) and C_16:0_. Based on polyphasic taxonomic analyses, strains CJ20^T^ and CJ99^T^ represent novel species of the genus *Sphingomonas*, for which names *Sphingomonas degradans* sp. nov. and *Sphingomonas paludis* are proposed, respectively. The type strains are CJ20^T^ (= KACC 23909 = JCM 37720) and CJ99^T^ (= KACC 24077 = JCM 37956).

## Introduction

The genus *Sphingomonas* was first described by Yabuuchi et al. (1990) and belongs to the family *Sphingomonadaceae* (Kosako et al., 2000). Large numbers of species previously classified within the genus *Sphingomonas* now have been reclassified into four genera, *Sphingomonas*, *Sphingobium*, *Novosphingobium*, and *Sphingopyxis*, on the basis of phylogenetic and chemotaxonomic analyses by Takeuchi et al. (2001). At the time of writing, the genus *Sphingomonas* comprises 181 species with validly published names listed in the List of Prokaryotic names with Standing in Nomenclature (Parte et al., 2020), with *S. paucimobilis* as the type species (Yabuuchi et al., 1990). Members of the genus are mainly found in the soil environment (Choi et al., 2024; Yang et al., 2006) but have been isolated from a wide range of habitats, including in wastewater (Fujii et al., 2001; Yoon et al., 2009), marine crustacean (Romanenko et al., 2009), desert (Dong et al., 2022) and air (Kim et al., 2014a>). *Sphingomonas* species are also known for their beneficial roles in bioremediation (Chen et al., 2008) and plant growth promotion (Asaf et al., 2020). Common characteristics of the genus are Gram-negative, yellowish and rod-shaped, and strictly aerobic. The predominant isoprenoid quinone is ubiquinone (Q-10) and the predominant polar lipid is sphingoglycolipid (SGL). The DNA G + C content of *Sphingomonas* ranges from 62 to 68 mol% (Wang et al., 2019). In this study, strains CJ20^T^ and CJ99^T^ isolated from the Han River and a wetland in South Korea, respectively, were characterized based on the polyphasic taxonomy approach using *S. hengshuiensis* WHSC-8^T^ (Wei et al., 2015), *S. soli* T5-04^T^ (Yang et al., 2006), *S. kyeonggiensis* THG-DT81^T^ (Son et al., 2014), and *S. azotifigens* Y39^T^ (Xie and Yokota, 2006) as related type strains for comparison based on phylogenetic proximity.

## Materials and Methods

### Isolation of bacterial strains and culture conditions

Strain CJ20^T^ and CJ99^T^ were isolated from the surface water of the Han River (37° 29' 06.4" N 127° 29' 17.9" E) and a wetland in Ungok (35° 28' 27.5"N 126° 37' 59.2" E), South Korea, respectively. The isolates were cultured in R2A agar (BD) at 30℃ for two days and preserved in glycerol suspension (30%, w/v) at −80°C.

### 16S rRNA gene-based phylogenetic analysis

The 16S rRNA genes were amplified from a single colony by PCR using AccuPower PCR premix (Bioneer) and primers 27F and 1492R. 16S rRNA gene sequences were determined at Biofact using an automated DNA analyser (ABI 3730XL; Applied Biosystems) with sequencing primers 27F, 1492R, 518F, and 805R to generate consensus sequences (Baker et al., 2003). The assembled 16S rRNA gene sequence data were aligned with the related strains from the EzBioCloud (http://www.ezbiocloud.net) (Yoon et al., 2017) and NCBI database (http://www.ncbi.nlm.nih.gov) (Sayers et al., 2022), using the multiple sequence alignment program MUSCLE (Edgar, 2004). Phylogenetic trees were constructed using MEGA 11 software (Tamura et al., 2021) based on neighbor-joining (NJ) and maximum likelihood (ML) methods. The evolutionary distances of NJ tree were calculated using the Jukes-Cantor model (Jukes and Cantor, 1969). Accordingly, the best substitution model for the ML tree was determined by model test option of the MEGA 11 software. The evolutionary distances were calculated using the Kimura 2-parameter model (Srivathsan and Meier, 2012) with non-uniformity of evolutionary rates among sites by using a discrete Gamma distribution (+ G) with five rate categories and by assuming that a certain fraction of sites is evolutionarily invariable (+ I). All positions containing gaps and missing data were eliminated from the dataset (complete deletion option). Topology of trees was evaluated based on bootstrap analysis of 1000 datasets.

### Whole genome sequencing and de novo assembly

The genomic DNA was extracted using the DNeasy^®^ Blood & Tissue kit (Qiagen) following the manufacturer’s instructions. Whole genome sequencing (WGS) of strains CJ20^T^ and CJ99^T^ were carried out at DNA LINK Inc. (Korea) using a hybrid assembly approach of the Oxford Nanopore MinION flow cell R9.4.1 FLO-MIN106 (Oxford Nanopore) for strain CJ20^T^ and R10.4.1 FLO-MIN114 (Oxford Nanopore) for strain CJ99^T^, and the Illumina NovaSeq 6000 sequencing platforms. The genomic DNA library construction for the nanopore sequencing was performed according to the Ligation Sequencing Kit SQK-LSK109 (Oxford Nanopore) for strain CJ20^T^ and SQK-LSK114 (Oxford Nanopore) for strain CJ99^T^ following manufacturer’s instructions. The genomic DNA library for the Illumina sequencing was prepared using TruSeq DNA PCR-Free kit according to manufacturer’s instructions. Genome hybrid assembly was performed using the Unicycler v0.5.0 (Wick et al., 2017) for strain CJ20^T^ and Hybracter v0.11.2 (Bouras et al., 2024). The assembled genome of both strains was evaluated using the BUSCO software according to default parameters and using single-copy gene datasets of *Sphingomonadales* lineages v5.7.1 (Manni et al., 2021).

### Phylogenomic and genomic analyses

According to the minimum standard for the use of genome data for the taxonomic study of prokaryotes proposed by Chun et al. (2018), the digital DNA-DNA hybridization (dDDH) values were calculated using Genome-to-Genome Distance Calculator (GGDC 3.0) (Meier-Kolthoff et al., 2022) and the average nucleotide identity (ANI) values were calculated by CJ Bioscience’s online ANI calculator Orthologous Average Nucleotide Identity Tool (OAT) with OrthoANI algorithm (Lee et al., 2016). The up-to-date bacterial core-gene 2 (UBCG2) and the UBCG2 phylogenomic pipeline (Kim et al., 2021) were used to generate the 81 UBCGs alignment sequences. The phylogenomic tree based on the concatenated sequences was constructed by IQ-TREE v.2.4.0 using the general time reversible substitution model with invariable sites and four gamma categories (Minh et al., 2020). Reference genome sequences were downloaded from the EzBioCloud (http://www.ezbiocloud.net) (Yoon et al., 2017) and NCBI genome database (http://www.ncbi.nlm.nih.gov) (Sayers et al., 2022). Protein-coding genes (CDS), rRNA, and tRNA genes were predicted using Bakta v1.9.1 (Schwengers et al., 2021). Antibiotic resistance genes were identified using the Comprehensive Antibiotic Resistance Database (CARD) (Alcock et al., 2023). Functional genes and their respective pathways within the genome were identified using BlastKOALA with KEGG database (Kanehisa et al., 2016). Pan- and core-genome analyses were performed using Panaroo v1.5.2 (Tonkin-Hill et al., 2020) and visualized in R using package venn (Dusa, 2024). The functional metabolic pathways, carbohydrate-active enzymes, and proteolytic enzymes were predicted using the KEGG, CAZy, MEROPS databases, respectively, implemented in METABOLIC software (version 4.0) (Zhou et al., 2022).

### Morphological and physiological characterization

Cellular morphology of strains CJ20^T^ and CJ99^T^ were observed by transmission electron microscopy using cells grown for 2 days at 30℃ on TSA. Growth of both strains was tested at 30℃ for 48 h on Reasoner’s 2A agar (R2A), Nutrient agar (NA), Luria-Bertani (LB) agar, Marine agar (MA), and Tryptic Soy Agar (TSA). Optimal temperature for growth was determined on TSA at 4, 10, 20, 30, 37, 40, and 45℃, and optimal pH for growth was determined in tryptic soy broth (TSB) using 0.1 M citrate buffer for pH 4–5, 0.1 M phosphate buffer for pH 6–8, and 0.1 M carbonate/bicarbonate for pH 9. Halotolerance test was conducted in TSB supplemented with 0–4% (w/v) NaCl (at 1% interval). Anaerobic growth was determined after two weeks of cultivation at 30℃ on TSA using the GasPak Anaerobic Pouch System (BD). Cell motility was tested by incubating bacterial strains in semi-solid tryptic soy medium containing 0.4% agar. The flexirubin-type pigment test was carried out by placing colonies in 20% (w/v) KOH solution and interpreting changes in colony color. Hydrolysis of starch, cellulose, casein and DNA was tested using 1.0% (w/v) soluble starch (BD), 0.2% (w/v) carboxymethylcellulose (Sigma Aldrich), 3% (w/v) skimmed milk (BD), and DNA test agar (BD), respectively. After two days of incubation, hydrolysis of starch and cellulose was visualized by iodine, and casein and DNA were confirmed by the transparency of the medium. Oxidase and catalase activities were performed using the oxidase reagent (bioMérieux) and 3% (v/v) aqueous H_2_O_2_ solution, respectively. Assimilation and enzyme activity tests were performed using API 20NE according to the manufacturer’s instructions (bioMérieux).

### Chemotaxonomic analysis

The polar lipids of strain CJ20^T^ and CJ99^T^ were extracted from 100 mg of freeze-dried cells cultured in TSA during two days at 30°C and analyzed by two-dimensional silica gel thin-layer chromatography (TLC) (Minnikin et al., 1984). Two types of solvents were used for two-dimensional TLC: a mixture of chloroform:methanol:water (32.5:12.5:2, v/v/v) was used for the first dimension of separation and a mixture of chloroform:methanol:acetic acid:water (40:6:7.5:2, v/v/v/v) was used for the second dimension. Total lipids were detected with 5% (w/v) phosphomolybdic acid, aminolipids with 0.25% (w/v) ninhydrin, phospholipids with molybdenum blue spray and glycolipids with 15% (w/v) α-naphthol. Respiratory quinone was extracted as described by Minnikin et al. (1984) and analyzed by high-performance liquid chromatography using a reverse phase column (Apollo C18, 250 × 4.6 mm, 5 μm) (Collins, 1985). Respiratory quinones were dissolved in chloroform:methanol (2:1, v/v) and eluted by a mixture of methanol:2-propanol (1:1, v/v) with a flow rate of 1 ml/min and detected at 275 nm. To extract fatty acids, both strains were incubated in TSB at 30℃ and harvested at the mid-exponential growth phase. The harvested cells were saponified, methylated, and extracted and analyzed by gas chromatography following the instructions of the Sherlock Microbial Identification System (MIDI) version 6.2 and identified by using the RTSBA6 database (Sasser, 1990).

### Antimicrobial susceptibility test

Antimicrobial susceptibility tests of strains CJ20^T^ and CJ99^T^ were performed by disk diffusion assay using commercial disks (Liofilchem, Italy) with amoxicillin (30 μg), cephalexin (30 μg), chloramphenicol (30 μg), clindamycin (2 μg), ciprofloxacin (5 μg), erythromycin (15 μg), fosfomycin (200 μg), gentamicin (10 μg), meropenem (10 μg), rifampicin (5 μg), streptomycin (10 μg), and tetracycline (30 μg). The susceptibility results were specified as S (susceptibility) and R (resistance) based on *Escherichia coli* ATCC 25922 and *Staphylococcus aureus* ATCC 29213 EUCAST clinical breakpoints (version 15.0).

### GenBank accession numbers

The GenBank accession numbers for the 16S rRNA gene sequences of strains CJ20^T^ and CJ99^T^ are PQ474648 and JQ809435, respectively, and the accession numbers for the genome sequences are CP177172 and CP187558, respectively.

## Results and Discussion

### 16S rRNA phylogeny

**Fig. 1.**
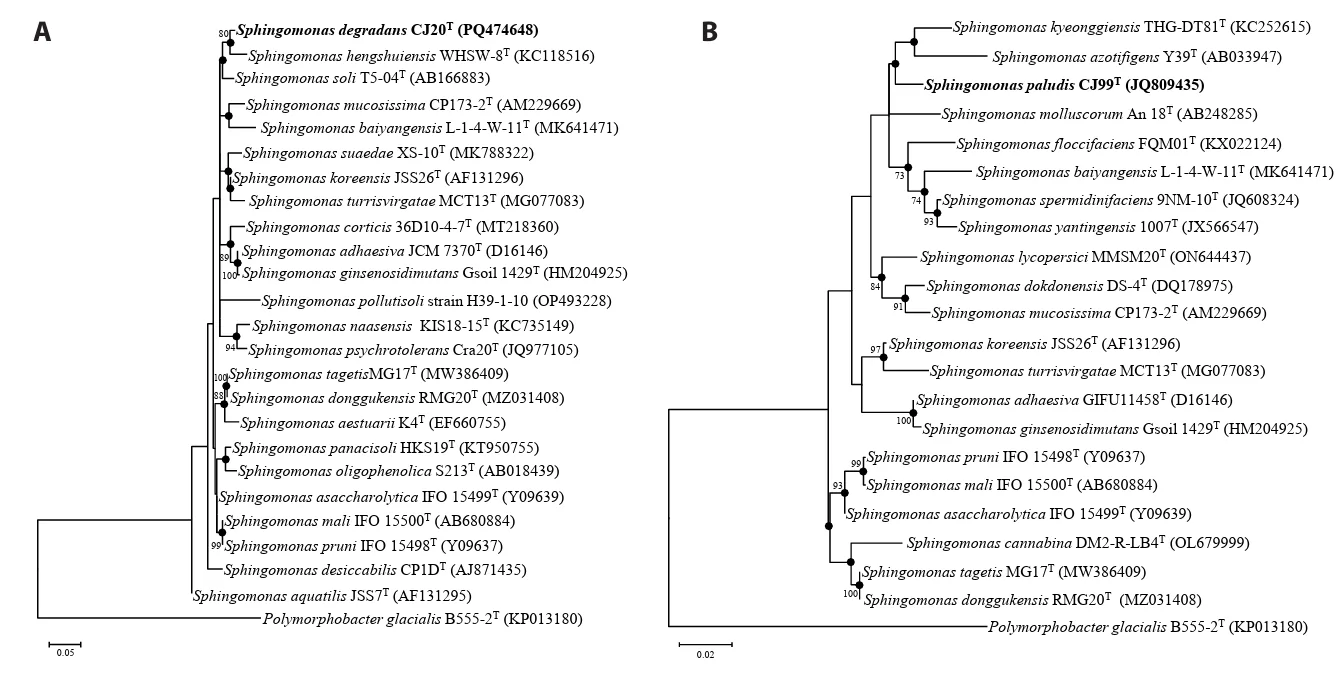
Maximum-likelihood phylogenetic tree of two novel species and related type strains based on 16S rRNA gene sequence. Strain CJ20^T^ and related type strains are shown in (A) and strain CJ99^T^, and related type strains are shown in (B). Closed circles indicate nodes recovered in trees generated by neighbor-joining and maximum-likelihood methods. Bootstrap values greater than 70% are shown at branch points based on maximum-likelihood analysis of 1,000 replicated datasets. *Polymorphobacter glacialis* B555-2^T^ was used as an outgroup. Bar, 0.05 (A) and 0.02 (B) substitutions per nucleotide position.

Analysis of 16S rRNA gene sequences revealed that strain CJ20^T^ had the highest sequence similarity with *S. asaccharolytica* Y-345^T^ (97.87%) and strain CJ99^T^ with *S. koreensis* JSS26^T^ (97.58%). Phylogenetic tree indicated that strain CJ20^T^ formed a robust clade with *S. hengshuiensis* WHSC-8^T^ and strain CJ99^T^ formed a distinct phylogenetic lineage forming a clade with *S. kyeonggiensis* THG-DT81^T^ and *S. azotifigens* Y39^T^ within the genus *Sphingomonas* (Fig. 1). Based on the pairwise 16S rRNA gene sequence identities below the established threshold for species demarcation (Kim et al., 2014b; Rosselló-Móra and Amann, 2015), strains CJ20^T^ and CJ99^T^ represent novel species within the genus *Sphingomonas*.

### Genomic features

**Fig. 2.**
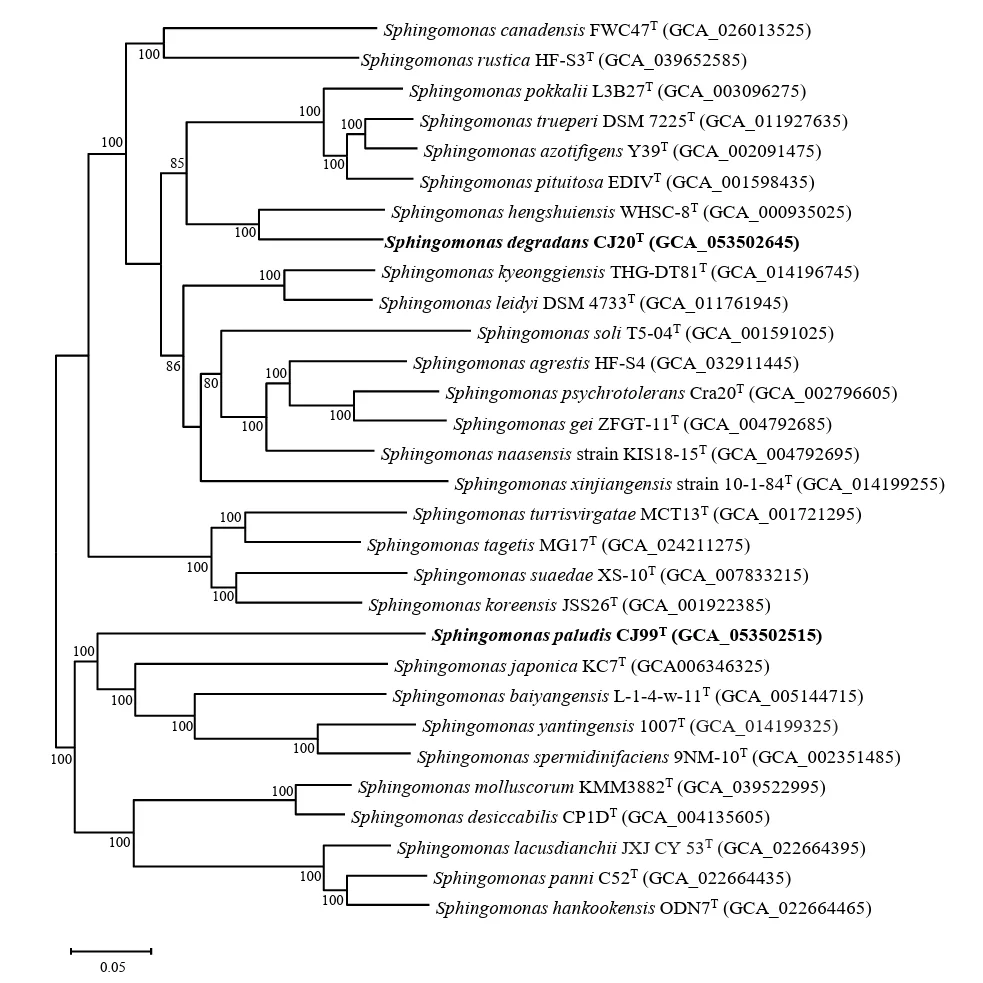
Phylogenomic tree of two novel species and type strains of the genus *Sphingomonas* based on 81 UBCGs. This tree is a part of the phylogenomic tree including the entire species of *Sphingomonas*. Bootstrap values greater than 70% are shown at branch points based on maximum-likelihood analysis of 1,000 replicated datasets. *Polymorphobacter* B555-2^T^ was used as an outgroup. Bar, 0.05 substitutions per nucleotide position.

The completeness of the genome assembly of strains CJ20^T^ and CJ99^T^ was evaluated to be 99.70% and 99.51% by BUSCO analysis, respectively (Fig. S3). WGS analysis revealed that strain CJ20^T^ had two identical copies of 16S rRNA gene, while CJ99^T^ had a single copy. Phylogenomic analysis showed that strain CJ20^T^ was most closely related to *S. hengshuiensis* WHSC-8^T^ and strain CJ99^T^ was most closely related to *S. japonica* KC7^T^ (Fig. 2). The genome size of strains CJ20^T^ and CJ99^T^ were 4.63 Mb and 3.17 Mb, respectively. The G + C contents of strains CJ20^T^ and CJ99^T^ were 67.0% and 65.8%, respectively. These results are consistent with the reported G + C content range of 62 to 68% for the genus *Sphingomonas* (Wang et al., 2019). The predicted genes of strain CJ20^T^ included 4,183 CDS, 6 rRNA genes, and 52 tRNA genes. The genome of strain CJ99^T^ consisted of 2,991 CDS, 3 rRNA, and 46 tRNA genes. The genomic characteristics of strains CJ20^T^ and CJ99^T^ and related type strains are presented in Table 1. To analyze the overall genomic similarity of strains CJ20^T^ and CJ99^T^ with their related type strains, ANI and dDDH values were calculated (Table 2). The ANI values of strain CJ20^T^ with *S. hengshuiensis* WHSC-8^T^, *S. pokkalii* L3B27T^T^, *S. azotifigens* Y39^T^, and *S. pituitosa* EDIV^T^ showed 82.92%, 78.06%, 78.32%, and 78.35%, respectively. The ANI values of strain CJ99^T^ with *S. japonica* KC7^T^, *S. baiyangensis* L-1-4-w-11^T^, and *S. yantingensis* 1007^T^ were 74.31%, 74.26%, and 74.10%, respectively. The dDDH values between strain CJ20^T^ and *S. hengshuiensis* WHSC-8^T^, *S. pokkalii* L3B27T^T^, *S. azotifigens* Y39^T^, and *S. pituitosa* EDIV^T^ were 34.4%, 20.9%, 22.0%, and 22.3%, respectively; the values between strain CJ99^T^ and *S. japonica* KC7^T^, *S. baiyangensis* L-1-4-w-11^T^, and *S. yantingensis* 1007^T^ were 16.8%, 16.1%, and 16.2%, respectively. Based on the ANI values below the threshold (< 95–96%) and the dDDH values below 70%, strains CJ20^T^ and CJ99^T^ represent a novel species of the genus *Sphingomonas* at the genomic level (Chun et al., 2018; Richter and Rosselló-Móra, 2009). Antibiotic resistance genes (ARGs) were identified using the resistance gene identifier (RGI) based on the Comprehensive Antibiotic Resistance Database (CARD), which revealed the presence of the *adeF* gene (resistance-nodulation-cell division antibiotic efflux pump family) in both strains. Functional gene annotation using BlastKOALA identified the *bla* gene (*β*-lactam resistance gene) in both strains. Additionally, strain CJ20^T^ had xenobiotic biodegradation pathways such as benzoate, catechol, and xylene. These results were consistent with the phenotypic resistance to the *β*-lactam antibiotic meropenem. Both strains showed inconsistency between their phenotypes and genotypes, indicating the presence of unreported ARGs (Fig. S4). Analysis of functional metabolic pathways revealed that CJ20^T^ and CJ99^T^ had metabolic profiles similar to those of the related type strains. Both strains encoded a number of carbohydrate-active enzymes, including glycoside hydrolases and polysaccharide lyases. The protease profiles of both strains and their related type strains were also similar, with serine and metalloprotease enzymes being predominant (Fig. S5). Venn diagram of strain CJ20^T^ and CJ99^T^ drawn with related type strains showed that each strain had 1,341 and 271 core genes, respectively (Fig. S6).

### Morphological, physiological, and biochemical characteristics

Cells of strains CJ20^T^ and CJ99^T^ were Gram-stain-negative, strictly aerobic, and yellow-colored colonies. Strain CJ20^T^ was non-motile, whereas strain CJ99^T^ was motile. Transmission electron micrographs showed that both cells were short rod-shaped (Fig. S7). The optimum growth medium was TSA and no growth was observed on MA. Growth of strain CJ20^T^ occurred at 10–37℃ with an optimum temperature of 30℃, while strain CJ99^T^ grew at 10–40℃ with an optimum temperature of 30℃. Strain CJ20^T^ grew at pH 6–8 (optimum pH 7) in the presence of 0–3% NaCl (optimum 0%). Strain CJ99^T^ grew at pH 7–8 (optimum pH 7) in the presence of 0–2% NaCl (optimum 0%). Both strains did not produce flexirubin-type pigments. The differential biochemical and physiological characteristics of strains CJ20^T^ and CJ99^T^ with closely related type strains are presented in Table 3.

### Chemotaxonomic characteristics

The polar lipids of strain CJ20^T^ contained phosphatidylethanolamine (PE), diphosphatidylglycerol (DPG), phosphatidylglycerol (PG), SGL, one unidentified phospholipid, and one unidentified lipid; those of strain CJ99^T^ included PE, DPG, PG, SGL, two unidentified phosphoglycolipids, and three unidentified lipids (Fig. S8). Both strains shared the same major polar lipids with most members of the genus *Sphingomonas*. The respiratory quinone of both strains was ubiquinone (Q-10), which coincides with other species of the genus *Sphingomonas*. The major fatty acids of both strains, including summed feature 8 (C_18:1_ *ω7c* and/or C_18:1_ *ω6c*) and C_16:0_, were concordant with their related type strains. The presence of C_8:0_ 3-OH and the absence of C_17:1_ *ω6c* in CJ20^T^ was distinguished from its related species, while the presence of C_17:0_, C_16:1_ *ω5c*, and C_17:1_ *ω8c* in CJ99^T^ was distinguished from other related species. The fatty acid profiles of strains CJ20^T^ and CJ99^T^ are summarized in Table 4 along with the related type strains.

### Taxonomic conclusion

Based on the results from the phylogenetic analysis, strains CJ20^T^ and CJ99^T^ belong to the genus *Sphingomonas*. Chemotaxonomic and phenotypic characteristics, including respiratory quinone, profiles of polar lipid and fatty acid, and carbon source utilization, were concordant with the traits listed in the genus description. Some differential characteristics distinguished strains CJ20^T^ and CJ99^T^ from their related type strains. Strain CJ20^T^ was positive for assimilation of *N*-acetyl-glucosamine, whereas its related type strains were negative. Strain CJ99^T^ assimilated potassium gluconate and adipic acid, while its related type strains did not. For the genomic analysis, low dDDH (< 70%) and ANI values (< 95–96%) precisely differentiated strains CJ20^T^ and CJ99^T^ from their closely related type strains. Therefore, strains CJ20^T^ and CJ99^T^ represent a novel species of the genus *Sphingomonas*, for which the names *Sphingomonas degradans* sp. nov. and *Sphingomonas paludis* sp. nov. are proposed, respectively.

### Description of *Sphingomonas degradans* sp. nov.

*Sphingomonas degradans* (de.gra’dans. L. part. adj. *degradans*, returning to the original order, referring to the ability of the type strain to degrade xenobiotic compounds).

Cells are aerobic, Gram-stain-negative, non-motile and rod-shaped. Colonies grown on TSA are circular and yellow-colored. Growth occurs at 10–37℃ (optimum, 30℃), pH 6–8 (optimum, pH 7), and 0–3% NaCl (optimum, 0%). Catalase activity is found to be positive but oxidase is negative. Esculin ferric citrate is hydrolyzed but DNA, casein, starch, cellulose, and gelatin are not. Flexirubin-type pigments are not produced. Nitrate and nitrite are not reduced. Indole production is not detected. ᴅ-Glucose is not fermented. Enzyme activity of *β*-galactosidase is positive but urease and arginine dihydrolase are negative. According to the API 20NE test, ᴅ-glucose, ʟ-arabinose, ᴅ-mannose, *N*-acetyl-glucosamine, ᴅ-maltose, and malic acid are assimilated, but D-mannitol, potassium gluconate, capric acid, adipic acid, trisodium citrate, and phenylacetic acid are not. The predominant polar lipids are phosphatidylethanolamine, diphosphatidylglycerol, phosphatidylglycerol, sphingoglycolipid, one unidentified phospholipid, and one unidentified lipid. The major fatty acids are summed feature 8 (C_18:1_ *ω7c* and/or C_18:1_ *ω6c*) and C_16:0_. The respiratory quinone is ubiquinone (Q-10). The DNA G + C content of the type strain is 67.0%. The type strain is CJ20^T^ (= KACC 23909 = JCM 37720) isolated from the Han River in Seoul, Korea.

### Description of *Sphingomonas paludis* sp. nov.

*Sphingomonas paludis* (pa.lu'dis. L. gen. n. *paludis*, of a swamp, of a wetland).

Cells are aerobic, Gram-stain-negative, motile and rod-shaped. Colonies grown on TSA are circular and yellow-colored. Growth occurs at 10–40℃ (optimum, 30℃), pH 7–8 (optimum pH, 7), and 0–2% NaCl (optimum, 0%). Catalase and oxidase activity are found to be positive. Hydrolysis of Esculin ferric citrate, DNA, casein, starch, and cellulose are positive but gelatin is not. Flexirubin-type pigments are not produced. Nitrate and nitrite are not reduced. Indole production is not detected. D-Glucose is not fermented. Enzyme activity of *β*-galactosidase is positive but urease and arginine dihydrolase are negative. According to the API 20NE test, ᴅ-glucose, ʟ-arabinose, ᴅ-mannose, ᴅ-maltose, potassium gluconate, adipic acid, and malic acid are assimilated, but ᴅ-mannitol, *N*-acetyl-glucosamine, capric acid, trisodium citrate, and phenylacetic acid are not. The predominant polar lipids are phosphatidylethanolamine, diphosphatidylglycerol, phosphatidylglycerol, sphingoglycolipid, three unidentified lipid, and two unidentified phosphoglycolipid. The major fatty acids are summed feature 8 (C_18:1_ *ω7c* and/or C_18:1_ *ω6c*) and C_16:0_. The respiratory quinone is ubiquinone (Q-10). The DNA G + C content of the type strain is 65.8%. The type strain is CJ99^T^ (=KACC 24077 = JCM 37956) isolated from a wetland in Ungok, Korea.

## Figures and Tables

**Table 1.** Genomic features of strains CJ20^T^, CJ99^T^ and related type strains Strains: 1, *S. degradans* CJ20^T^; 2, *S. hengshuiensis* WHSC-8^T^; 3, *S. pokkalii* L3B27T^T^; 4, *S. azotifigens* Y39^T^; 5, *S. pituitosa* EDIV^T^; 6, *S. paludis* CJ99^T^; 7, *S. japonica* KC7^T^; 8, *S. baiyangensis* L-1-4-w-11^T^; 9, *S. yantingensis* 1007^T^.

Genome feature	1	2	3	4	5	6	7	8	9
Size (Mb)	4.63	4.8	4.2	5.1	4.7	3.17	2.9	3.3	3.6
G + C (%)	67.0	66.7	66	67.5	67	65.8	66.5	68.0	68.0
CDS	4183	4784	3846	4656	4340	2991	2784	3153	3430
Contigs	2	2	34	163	75	1	1	7	11
rRNA genes	6	7	3	3	5	3	3	3	3
tRNA genes	52	49	48	44	46	46	46	47	47

**Table 2.** ANI and dDDH values between strains CJ20^T^, CJ99^T^ and related type strains Strains: 1, *S. degradans* CJ20^T^; 2, *S. hengshuiensis* WHSC-8^T^; 3, *S. pokkalii* L3B27T^T^; 4, *S. azotifigens* Y39^T^; 5, *S. pituitosa* EDIV^T^; 6, *S. paludis* CJ99^T^; 7, *S. japonica* KC7^T^; 8, *S. baiyangensis* L-1-4-w-11^T^; 9, *S. yantingensis* 1007^T^.

dDDH	1	2	3	4	5	6	7	8	9
ANI									
1		34.4	20.9	22.0	22.3	16.1	17.4	17.5	17.3
2	82.9		19.5	20.5	20.6	15.5	17.2	17.8	17.1
3	78.1	77.3		40.2	36.9	15.0	16.4	16.6	16.6
4	78.3	77.7	85.1		43.4	14.8	16.5	16.7	16.9
5	78.4	78.0	84.7	87.1		15.1	16.6	17.1	17.3
6	74.4	74.0	73.7	73.9	73.9		16.8	16.1	16.2
7	75.3	75.5	74.5	75.0	74.9	74.3		23.8	20.7
8	75.3	75.3	74.6	75.2	74.9	74.3	76.8		25.2
9	75.0	75.0	74.2	74.8	74.8	74.1	76.2	77.9	

**Table 3.** Differential characteristics of strains CJ20^T^, CJ99^T^ and related type strains of the genus *Sphingomonas* Strains: 1, *S. degradans* CJ20^T^; 2, *S. hengshuiensis* WHSC-8^T^; 3, *S. soli* T5-04^T^; 4, *S. paludis* CJ99^T^; 5, *S. kyeonggiensis* THG-DT81^T^; 6, *S. azotifigens* Y39^T^; +, positive; −, negative.

Characteristics	1	2	3	4	5	6
Isolation source*	Han River	Hengshui Lake^a^	Ginseng field^b^	Wetland	Ginseng field^c^	Roots^d^
Motility	Non-motile	Motile^a^	Non-motile^b^	Motile	Motile^c^	Motile^d^
Colony color	Yellow	Yellow	Yellow	Yellow	Yellow	Yellow-Orange
**Growth**						
Temperature (°C)	10–37	20–35^a^	15–37^b^	10–40	15–30^c^	25–37^d^
pH	6.0–8.0	5.0–10.0^a^	6.8–7.5^b^	7.0–8.0	6.0–9.5^c^	ND
NaCl (%, w/v)	0–3	0–1^a^	ND	0–2	0–0.5^c^	0–2.5^d^
**Hydrolysis of**						
Starch	−	−	−	+	−	−
Cellulose	−	−	−	+	−	−
Casein	−	−	−	+	−	−
DNA	−	+	+	+	−	+
**Assimilation of**						
ᴅ-Mannose	+	+	−	+	+	+
ᴅ-Mannitol	−	+	−	−	−	−
*N*-Acetyl-glucosamine	+	−	−	−	+	+
ᴅ-Maltose	+	+	−	+	+	+
Potassium gluconate	−	+	−	+	−	−
Adipic acid	−	+	−	+	−	−
Malic acid	+	+	−	+	+	+
**Enzyme activity**						
Oxidase	−	+	+	+	+	+
ᴅ-Glucose fermentation	−	−	−	−	−	+
*β*-Galactosidase	+	+	+	+	−	+

All data are from this study unless indicated otherwise.

Data taken from ^a^Wei et al. (2015); ^b^Yang et al. (2006); ^c^Son et al. (2014); ^d^Xie and Yokota (2006).

**Table 4.** Fatty acid composition (%) of strains CJ20^T^, CJ99^T^ and related type strains of genus *Sphingomonas* Strains: 1, *S. degradans* CJ20^T^; 2, *S. hengshuiensis* WHSC-8^T^; 3, *S. soli* T5-04^T^; 4, *S. paludis* CJ99^T^; 5, *S. kyeonggiensis* THG-DT81^T^; 6, *S. azotifigens* Y39^T^; TR, trace amount (< 1%); –, not detected.

Fatty acid	1	2	3	4	5	6
**Saturated**						
*iso*–C_10:0_	−	5.3	−	1.6	9.0	5.0
C_16:0_	**15.2**	**13.6**	**18.9**	**17.5**	**21.2**	**24.2**
C_17:0_	−	−	−	1.4	−	−
C_18:0_	−	−	−	−	1.2	1.4
**Unsaturated**						
C_16:1 _*ω*5*c*	−	−	−	1.4	−	−
C_17:1_ *ω*6*c*	−	5.4	3.8	15.1	2.7	TR
C_17:1_ *ω*8*c*	−	−	−	2.5	−	−
C_18:1_ *ω*5*c*	−	−	−	−	−	1.1
11methyl–C_18:1_ *ω*7*c*	−	−	4.6	2.7	4.3	5.9
cyclo–C_19:0_ *ω*8*c*	−	−	−	−	−	TR
**Hydroxy**						
C_8:0 _3–OH	5.5	−	−	−	−	−
C_14:0_ 2–OH	3.7	7.2	−	2.0	4.7	4.5
Summed features^*^						
3	3.0	5.3	7.5	9.0	−	−
8	**71.4**	**61.5**	**64.5**	**44.6**	**55.8**	**55.1**

All data are obtained from this study. Major components (> 10.0%) are highlighted in bold.

* Summed features represent groups of two or three fatty acids that could not be separated by MIDI system. Summed feature 3 comprises C_16:1_ *ω6c* and/or C_16:1_ *ω7c*, summed feature 8 comprises C_18:1_ *ω6c* and/or C_18:1_ *ω7c*.

## Supplementary Information

Fig. S1. Neighbor-joining (NJ) tree based on 16S rRNA gene sequences. Strain CJ20^T^ and related type strains are shown in (A) and strain CJ99^T^ and related type strains are shown in (B). Bootstrap values greater than 70% are shown at branch points based on 1000 replicated datasets. *Polymorphobacter glacialis* B555-2^T^ was used as an outgroup. Bar, 0.01 substitutions per nucleotide position.

Fig. S2. Phylogenomic tree of two novel species and type strains of the genus *Sphingomonas* based on 81 UBCGs. Bootstrap values greater than 70% are shown at branch points based on maximum-likelihood analysis of 1,000 replicated datasets. The genus *Polymorphobacter glacialis* B555-2^T^ was used as an outgroup. Bar, 0.10 substitutions per nucleotide position.

Fig. S3. Evaluation of the completeness of assembly results of strains CJ20^T^ and CJ99^T^.

Fig. S4. Inhibition zone diameter (IZD) of *S. degradans* CJ20^T^ and *S. paludis* CJ99^T^. Test was determined by disk diffusion assay using 6 mm commercial disk. Susceptibility/Resistance results are presented as susceptible (S) or resistance (R) based on EUCAST breakpoints (version 15.0) for *Escherichia coli* ATCC 25922 and *Staphylococcus aureus* ATCC 29213. AML, Amoxicillin; MRP, Meropenem; CL, Cephalexin; CIP, Ciprofloxacin; CN, Gentamicin; CD, Clindamycin; E, Erythromycin; TE, Tetracycline; C, Chloramphenicol; FOS, Fosfomycin; RD, Rifampicin; S, Streptomycin.

Fig. S5. Metabolic comparison of CJ20^T^ and CJ99^T^ with related type strains: core metabolic modules (A), carbohydrate-active enzymes (B), and protease types (C).

Fig. S6. Venn diagram of orthologous genes of strains CJ20^T^, CJ99^T^ and related type strains. The numbers indicate the number of shared and unique genes between type strains.

Fig. S7. Transmission electron micrograph of strains CJ20^T^ (A) and CJ99^T^ (B).

Fig. S8. Two-dimensional TLC of the total polar lipids from strains CJ20^T^ (A) and CJ99^T^ (B). Abbreviations: PE, phosphatidylethanolamine; DPG, diphosphatidylglycerol; PG, phosphatidylglycerol; SGL, sphingoglycolipid; AL, amino lipid; PGL, phosphoglycolipid; PL, unidentified phospholipid; L, unidentified lipid.
