# Supplementary material for: Sphingomonas degradans sp. nov. and Sphingomonas paludis sp. nov., isolated from the Han River and a wetland in South Korea
Source: J Microbiol. 2026 Jan 31;64(1):e2510010. doi: 10.71150/jm.2510010 (PMC13576999; doi:10.71150/jm.2510010)
Supplement: Supplementary file 1 [file jm-2510010-Supplementary-Fig-S1.pdf]

Fig. S1.

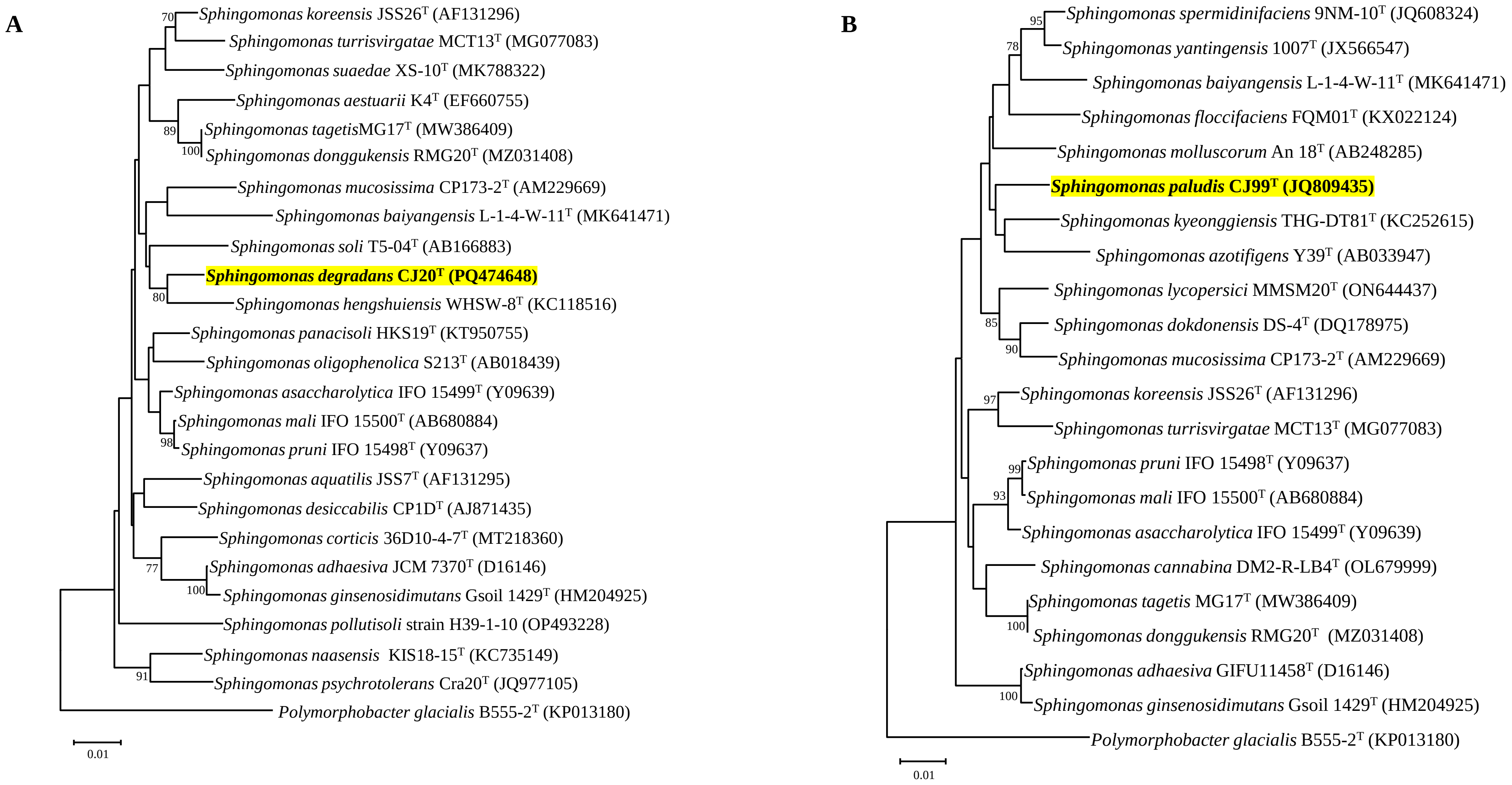

A
70
Sphingomonas koreensis JSS26T (AF131296)
Sphingomonas turrisvirgatae MCT13T (MG077083)
Sphingomonas suaedae XS-10T (MK788322)
Sphingomonas aestuarii K4T (EF660755)
89
Sphingomonas tagetisMG17T (MW386409)
100
Sphingomonas donggukensis RMG20T (MZ031408)
Sphingomonas mucosissima CP173-2T (AM229669)
Sphingomonas baiyangensis L-1-4-W-11T (MK641471)
Sphingomonas soli T5-04T (AB166883)
Sphingomonas degradans CJ20T (PQ474648)
80
Sphingomonas hengshuiensis WHSW-8T (KC118516)
Sphingomonas panacisoli HKS19T (KT950755)
Sphingomonas oligophenolica S213T (AB018439)
Sphingomonas asaccharolytica IFO 15499T (Y09639)
Sphingomonas mali IFO 15500T (AB680884)
98
Sphingomonas pruni IFO 15498T (Y09637)
Sphingomonas aquatilis JSS7T (AF131295)
Sphingomonas desiccabilis CP1DT (AJ871435)
Sphingomonas corticis 36D10-4-7T (MT218360)
77
Sphingomonas adhaesiva JCM 7370T (D16146)
100
Sphingomonas ginsenosidimutans Gsoil 1429T (HM204925)
Sphingomonas pollutisoli strain H39-1-10 (OP493228)
Sphingomonas naasensis KIS18-15T (KC735149)
91
Sphingomonas psychrotolerans Cra20T (JQ977105)
Polymorphobacter glacialis B555-2T (KP013180)
0.01
B
95
Sphingomonas spermidinifaciens 9NM-10T (JQ608324)
78
Sphingomonas yantingensis 1007T (JX566547)
Sphingomonas baiyangensis L-1-4-W-11T (MK641471)
Sphingomonas floccifaciens FQM01T (KX022124)
Sphingomonas molluscorum An 18T (AB248285)
Sphingomonas paludis CJ99T (JQ809435)
Sphingomonas kyeonggiensis THG-DT81T (KC252615)
Sphingomonas azotifigens Y39T (AB033947)
Sphingomonas lycopersici MMSM20T (ON644437)
85
Sphingomonas dokdonensis DS-4T (DQ178975)
90
Sphingomonas mucosissima CP173-2T (AM229669)
97
Sphingomonas koreensis JSS26T (AF131296)
Sphingomonas turrisvirgatae MCT13T (MG077083)
99
Sphingomonas pruni IFO 15498T (Y09637)
93
Sphingomonas mali IFO 15500T (AB680884)
Sphingomonas asaccharolytica IFO 15499T (Y09639)
Sphingomonas cannabina DM2-R-LB4T (OL679999)
Sphingomonas tagetis MG17T (MW386409)
100
Sphingomonas donggukensis RMG20T (MZ031408)
Sphingomonas adhaesiva GIFU11458T (D16146)
100
Sphingomonas ginsenosidimutans Gsoil 1429T (HM204925)
Polymorphobacter glacialis B555-2T (KP013180)
0.01
