# Supplementary material for: Sphingomonas degradans sp. nov. and Sphingomonas paludis sp. nov., isolated from the Han River and a wetland in South Korea
Source: J Microbiol. 2026 Jan 31;64(1):e2510010. doi: 10.71150/jm.2510010 (PMC13576999; doi:10.71150/jm.2510010)
Supplement: Supplementary file 2 [file jm-2510010-Supplementary-Fig-S2.pdf]

Fig. S2.

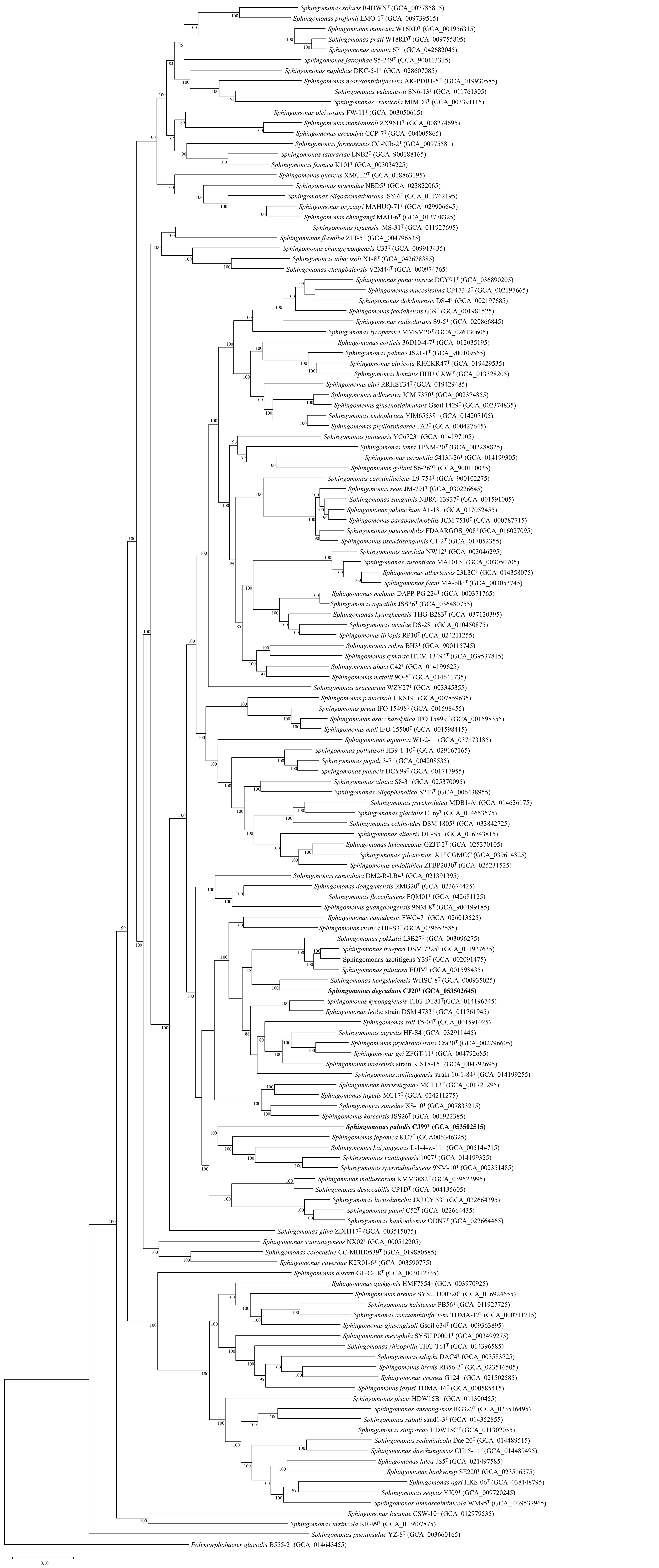
Sphingomonas solaris R4DWN^{T} (GCA_007785815)
Sphingomonas profundi LMO-1^{T} (GCA_009739515)
Sphingomonas montana W16RD^{T} (GCA_001956315)
Sphingomonas prati W18RD^{T} (GCA_009755805)
Sphingomonas arantia 6P^{T} (GCA_042682045)
Sphingomonas jatrophae S5-249^{T} (GCA_900113315)
Sphingomonas naphthae DKC-5-1^{T} (GCA_028607085)
Sphingomonas nostoxanthinifaciens AK-PDB1-5^{T} (GCA_019930585)
Sphingomonas vulcanisoli SN6-13^{T} (GCA_011761305)
Sphingomonas crusticola MIMD3^{T} (GCA_003391115)
Sphingomonas oleivorans FW-11^{T} (GCA_003050615)
Sphingomonas montanisoli ZX9611^{T} (GCA_008274695)
Sphingomonas crocodyli CCP-7^{T} (GCA_004005865)
Sphingomonas formosensis CC-Nfb-2^{T} (GCA_00975581)
Sphingomonas laterariae LNB2^{T} (GCA_900188165)
Sphingomonas fennica K101^{T} (GCA_003034225)
Sphingomonas quercus XMGL2^{T} (GCA_018863195)
Sphingomonas morindae NBD5^{T} (GCA_023822065)
Sphingomonas oligoaromativorans SY-6^{T} (GCA_011762195)
Sphingomonas oryzagri MAHUQ-71^{T} (GCA_029906645)
Sphingomonas chungangi MAH-6^{T} (GCA_013778325)
Sphingomonas jejuensis MS-31^{T} (GCA_011927695)
Sphingomonas flavalba ZLT-5^{T} (GCA_004796535)
Sphingomonas changnyeongensis C33^{T} (GCA_009913435)
Sphingomonas tabacisoli X1-8^{T} (GCA_042678385)
Sphingomonas changbaiensis V2M44^{T} (GCA_000974765)
Sphingomonas panaciterrae DCY91^{T} (GCA_036890205)
Sphingomonas mucosissima CP173-2^{T} (GCA_002197665)
Sphingomonas dokdonensis DS-4^{T} (GCA_002197685)
Sphingomonas jeddahensis G39^{T} (GCA_001981525)
Sphingomonas radiodurans S9-5^{T} (GCA_020866845)
Sphingomonas lycopersici MMSM20^{T} (GCA_026130605)
Sphingomonas corticis 36D10-4-7^{T} (GCA_012035195)
Sphingomonas palmae JS21-1^{T} (GCA_900109565)
Sphingomonas citricola RHCKR47^{T} (GCA_019429535)
Sphingomonas hominis HHU CXW^{T} (GCA_013328205)
Sphingomonas citri RRHST34^{T} (GCA_019429485)
Sphingomonas adhaesiva JCM 7370^{T} (GCA_002374855)
Sphingomonas ginsenosidimutans Gsoil 1429^{T} (GCA_002374835)
Sphingomonas endophytica YIM65538^{T} (GCA_014207105)
Sphingomonas phyllosphaerae FA2^{T} (GCA_000427645)
Sphingomonas jinjuensis YC6723^{T} (GCA_014197105)
Sphingomonas lenta 1PNM-20^{T} (GCA_002288825)
Sphingomonas aerophila 5413J-26^{T} (GCA_014199305)
Sphingomonas gellani S6-262^{T} (GCA_900110035)
Sphingomonas carotinifaciens L9-754^{T} (GCA_900102275)
Sphingomonas zeae JM-791^{T} (GCA_030226645)
Sphingomonas sanguinis NBRC 13937^{T} (GCA_001591005)
Sphingomonas yabuuchiae A1-18^{T} (GCA_017052455)
Sphingomonas parapaucimobilis JCM 7510^{T} (GCA_000787715)
Sphingomonas paucimobilis FDAARGOS_908^{T} (GCA_016027095)
Sphingomonas pseudosanguinis G1-2^{T} (GCA_017052355)
Sphingomonas aerolata NW12^{T} (GCA_003046295)
Sphingomonas aurantiaca MA101b^{T} (GCA_003050705)
Sphingomonas albertensis 23L3C^{T} (GCA_014358075)
Sphingomonas faeni MA-olki^{T} (GCA_003053745)
Sphingomonas melonis DAPP-PG 224^{T} (GCA_000371765)
Sphingomonas aquatilis JSS26^{T} (GCA_036480755)
Sphingomonas kyungheensis THG-B283^{T} (GCA_037120395)
Sphingomonas insulae DS-28^{T} (GCA_010450875)
Sphingomonas liriopis RP10^{T} (GCA_024211255)
Sphingomonas rubra BH3^{T} (GCA_900115745)
Sphingomonas cynarae ITEM 13494^{T} (GCA_039537815)
Sphingomonas abaci C42^{T} (GCA_014199625)
Sphingomonas metalli 9O-5^{T} (GCA_014641735)
Sphingomonas aracearum WZY27^{T} (GCA_003345355)
Sphingomonas panacisoli HKS19^{T} (GCA_007859635)
Sphingomonas pruni IFO 15498^{T} (GCA_001598455)
Sphingomonas asaccharolytica IFO 15499^{T} (GCA_001598355)
Sphingomonas mali IFO 15500^{T} (GCA_001598415)
Sphingomonas aquatica W1-2-1^{T} (GCA_037173185)
Sphingomonas pollutisoli H39-1-10^{T} (GCA_029167165)
Sphingomonas populi 3-7^{T} (GCA_004208535)
Sphingomonas panacis DCY99^{T} (GCA_001717955)
Sphingomonas alpina S8-3^{T} (GCA_025370095)
Sphingomonas oligophenolica S213^{T} (GCA_006438955)
Sphingomonas psychrolutea MDB1-A^{T} (GCA_014636175)
Sphingomonas glacialis C16y^{T} (GCA_014653575)
Sphingomonas echinoides DSM 1805^{T} (GCA_033842725)
Sphingomonas aliaeris DH-S5^{T} (GCA_016743815)
Sphingomonas hylomeconis GZJT-2^{T} (GCA_025370105)
Sphingomonas qilianensis X1^{T} CGMCC (GCA_039614825)
Sphingomonas endolithica ZFBP2030^{T} (GCA_025231525)
Sphingomonas cannabina DM2-R-LB4^{T} (GCA_021391395)
Sphingomonas donggukensis RMG20^{T} (GCA_023674425)
Sphingomonas floccifaciens FQM01^{T} (GCA_042681125)
Sphingomonas guangdongensis 9NM-8^{T} (GCA_900199185)
Sphingomonas canadensis FWC47^{T} (GCA_026013525)
Sphingomonas rustica HF-S3^{T} (GCA_039652585)
Sphingomonas pokkalii L3B27^{T} (GCA_003096275)
Sphingomonas trueperi DSM 7225^{T} (GCA_011927635)
Sphingomonas azotifigens Y39^{T} (GCA_002091475)
Sphingomonas pituitosa EDIV^{T} (GCA_001598435)
Sphingomonas hengshuiensis WHSC-8^{T} (GCA_000935025)
Sphingomonas degradans CJ20^{T} (GCA_053502645)
Sphingomonas kyeonggiensis THG-DT81^{T}(GCA_014196745)
Sphingomonas leidyi strain DSM 4733^{T} (GCA_011761945)
Sphingomonas soli T5-04^{T} (GCA_001591025)
Sphingomonas agrestis HF-S4 (GCA_032911445)
Sphingomonas psychrotolerans Cra20^{T} (GCA_002796605)
Sphingomonas gei ZFGT-11^{T} (GCA_004792685)
Sphingomonas naasensis strain KIS18-15^{T} (GCA_004792695)
Sphingomonas xinjiangensis strain 10-1-84^{T} (GCA_014199255)
Sphingomonas turrisvirgatae MCT13^{T} (GCA_001721295)
Sphingomonas tagetis MG17^{T} (GCA_024211275)
Sphingomonas suaedae XS-10^{T} (GCA_007833215)
Sphingomonas koreensis JSS26^{T} (GCA_001922385)
Sphingomonas paludis CJ99^{T} (GCA_053502515)
Sphingomonas japonica KC7^{T} (GCA006346325)
Sphingomonas baiyangensis L-1-4-w-11^{T} (GCA_005144715)
Sphingomonas yantingensis 1007^{T} (GCA_014199325)
Sphingomonas spermidinifaciens 9NM-10^{T} (GCA_002351485)
Sphingomonas molluscorum KMM3882^{T} (GCA_039522995)
Sphingomonas desiccabilis CP1D^{T} (GCA_004135605)
Sphingomonas lacusdianchii JXJ CY 53^{T} (GCA_022664395)
Sphingomonas panni C52^{T} (GCA_022664435)
Sphingomonas hankookensis ODN7^{T} (GCA_022664465)
Sphingomonas gilva ZDH117^{T} (GCA_003515075)
Sphingomonas sanxanigenens NX02^{T} (GCA_000512205)
Sphingomonas colocasiae CC-MHH0539^{T} (GCA_019880585)
Sphingomonas cavernae K2R01-6^{T} (GCA_003590775)
Sphingomonas deserti GL-C-18^{T} (GCA_003012735)
Sphingomonas ginkgonis HMF7854^{T} (GCA_003970925)
Sphingomonas arenae SYSU D00720^{T} (GCA_016924655)
Sphingomonas kaistensis PB56^{T} (GCA_011927725)
Sphingomonas astaxanthinifaciens TDMA-17^{T} (GCA_000711715)
Sphingomonas ginsengisoli Gsoil 634^{T} (GCA_009363895)
Sphingomonas mesophila SYSU P0001^{T} (GCA_003499275)
Sphingomonas rhizophila THG-T61^{T} (GCA_014396585)
Sphingomonas edaphi DAC4^{T} (GCA_003583725)
Sphingomonas brevis RB56-2^{T} (GCA_023516505)
Sphingomonas cremea G124^{T} (GCA_021502585)
Sphingomonas jaspsi TDMA-16^{T} (GCA_000585415)
Sphingomonas piscis HDW15B^{T} (GCA_011300455)
Sphingomonas anseongensis RG327^{T} (GCA_023516495)
Sphingomonas sabuli sand1-3^{T} (GCA_014352855)
Sphingomonas sinipercae HDW15C^{T} (GCA_011302055)
Sphingomonas sediminicola Dae 20^{T} (GCA_014489515)
Sphingomonas daechungensis CH15-11^{T} (GCA_014489495)
Sphingomonas lutea JS5^{T} (GCA_021497585)
Sphingomonas hankyongi SE220^{T} (GCA_023516575)
Sphingomonas agri HKS-06^{T} (GCA_038148795)
Sphingomonas segetis YJ09^{T} (GCA_009720245)
Sphingomonas limnosediminicola WM95^{T} (GCA_ 039537965)
Sphingomonas lacunae CSW-10^{T} (GCA_012979535)
Sphingomonas ursincola KR-99^{T} (GCA_013607875)
Sphingomonas paeninsulae YZ-8^{T} (GCA_003660165)
Polymorphobacter glacialis B555-2^{T} (GCA_014643455)
0.10
