# Supplementary material for: Sphingomonas degradans sp. nov. and Sphingomonas paludis sp. nov., isolated from the Han River and a wetland in South Korea
Source: J Microbiol. 2026 Jan 31;64(1):e2510010. doi: 10.71150/jm.2510010 (PMC13576999; doi:10.71150/jm.2510010)
Supplement: Supplementary file 3 [file jm-2510010-Supplementary-Fig-S3.pdf]

Fig S3.

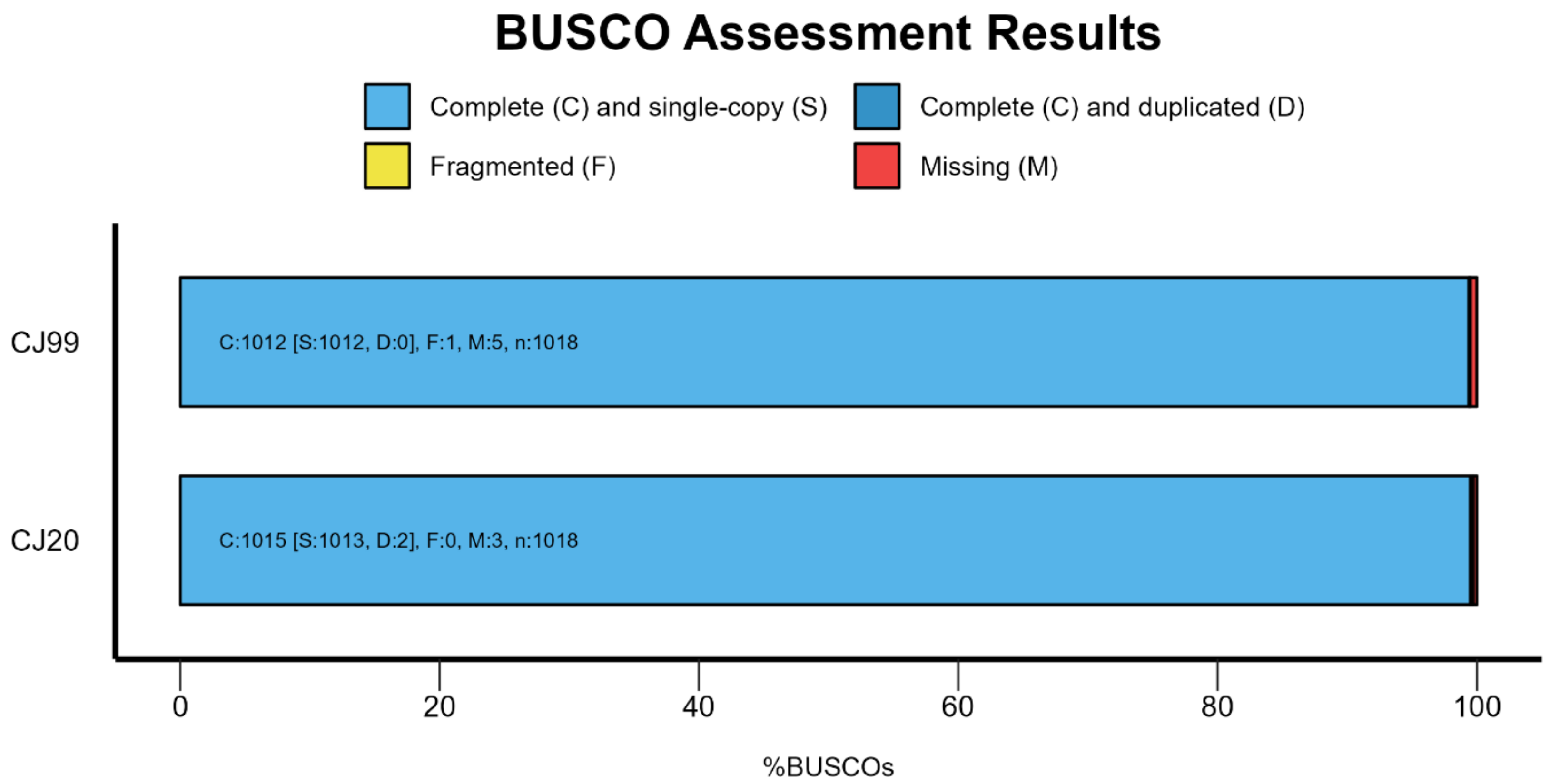
BUSCO Assessment Results
Complete (C) and single-copy (S)
Complete (C) and duplicated (D)
Fragmented (F)
Missing (M)
CJ99
C:1012 [S:1012, D:0], F:1, M:5, n:1018
CJ20
C:1015 [S:1013, D:2], F:0, M:3, n:1018
0
20
40
60
80
100
%BUSCOs
