# Supplementary material for: Sphingomonas degradans sp. nov. and Sphingomonas paludis sp. nov., isolated from the Han River and a wetland in South Korea
Source: J Microbiol. 2026 Jan 31;64(1):e2510010. doi: 10.71150/jm.2510010 (PMC13576999; doi:10.71150/jm.2510010)
Supplement: Supplementary file 4 [file jm-2510010-Supplementary-Fig-S4.pdf]

Fig S4.

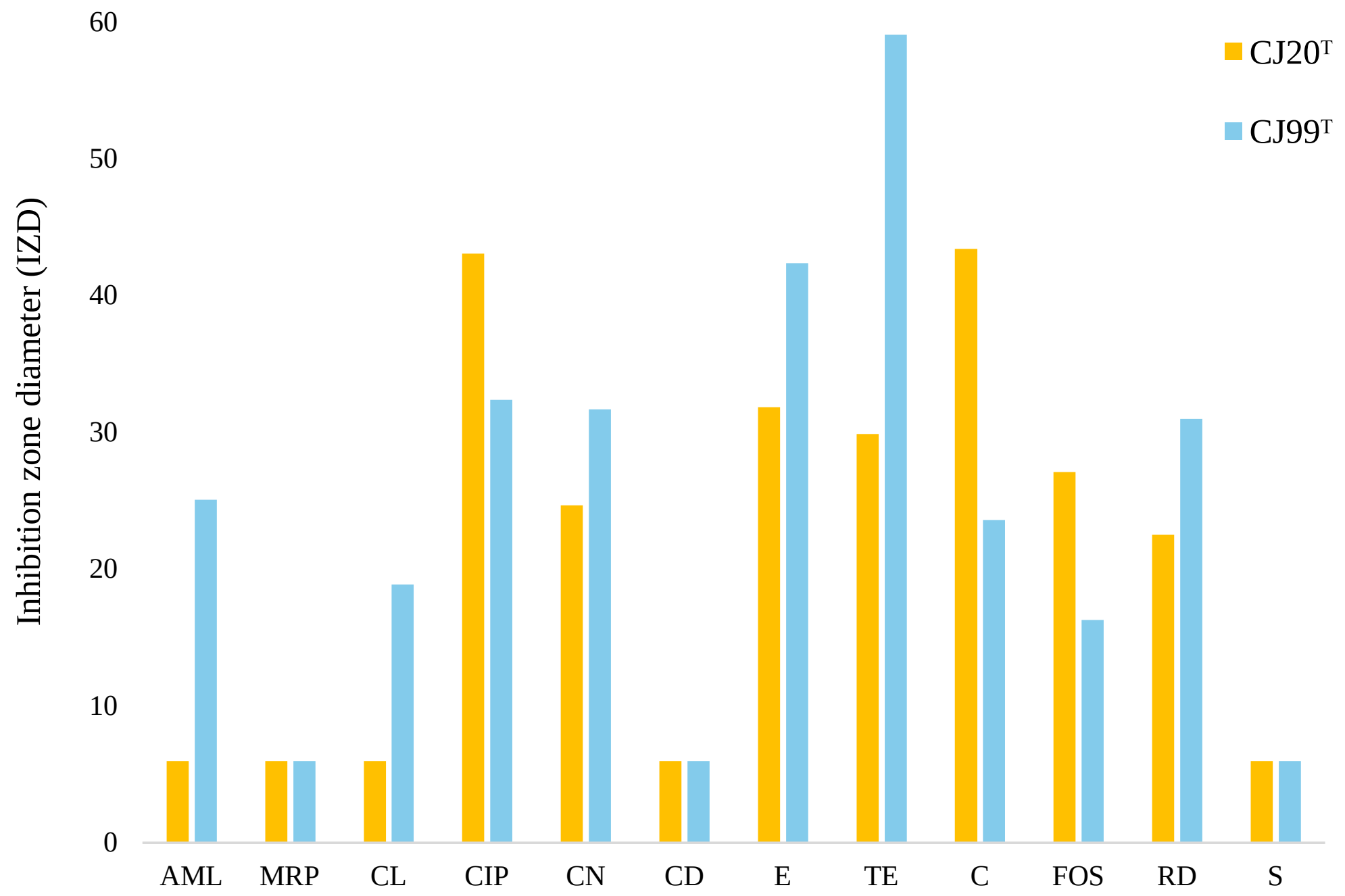

	AML	MRP	CL	CIP	CN	CD	E	TE	C	FOS	RD	S
CJ20^{T}	**R**	**R**	**R**	**S**	**S**	**R**	**S**	**S**	**S**	**S**	**R**	**R**
CJ99^{T}	**S**	**R**	**S**	**S**	**S**	**R**	**S**	**S**	**S**	**R**	**S**	**R**
