# Supplementary material for: Sphingomonas degradans sp. nov. and Sphingomonas paludis sp. nov., isolated from the Han River and a wetland in South Korea
Source: J Microbiol. 2026 Jan 31;64(1):e2510010. doi: 10.71150/jm.2510010 (PMC13576999; doi:10.71150/jm.2510010)
Supplement: Supplementary file 5 [file jm-2510010-Supplementary-Fig-S5.pdf]

Fig S5.

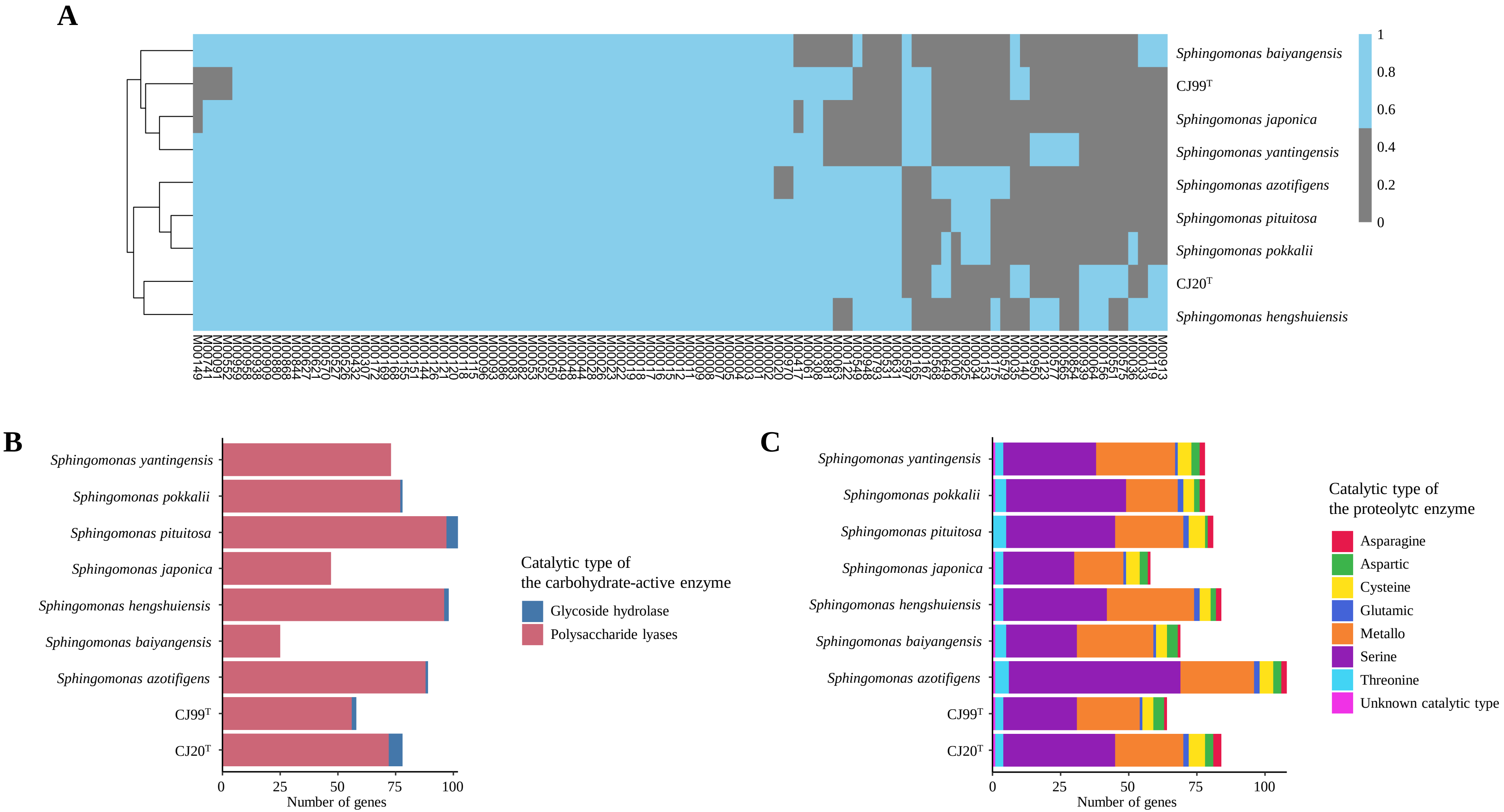
A
Sphingomonas baiyangensis
CJ99T
Sphingomonas japonica
Sphingomonas yantingensis
Sphingomonas azotifigens
Sphingomonas pituitosa
Sphingomonas pokkalii
CJ20T
Sphingomonas hengshuiensis
1
0.8
0.6
0.4
0.2
0
B
Sphingomonas yantingensis
Sphingomonas pokkalii
Sphingomonas pituitosa
Sphingomonas japonica
Sphingomonas hengshuiensis
Sphingomonas baiyangensis
Sphingomonas azotifigens
CJ99T
CJ20T
0
25
50
75
100
Number of genes
Catalytic type of
the carbohydrate-active enzyme
Glycoside hydrolase
Polysaccharide lyases
C
Sphingomonas yantingensis
Sphingomonas pokkalii
Sphingomonas pituitosa
Sphingomonas japonica
Sphingomonas hengshuiensis
Sphingomonas baiyangensis
Sphingomonas azotifigens
CJ99T
CJ20T
0
25
50
75
100
Number of genes
Catalytic type of
the proteolytc enzyme
Asparagine
Aspartic
Cysteine
Glutamic
Metallo
Serine
Threonine
Unknown catalytic type
