# Supplementary material for: Sphingomonas degradans sp. nov. and Sphingomonas paludis sp. nov., isolated from the Han River and a wetland in South Korea
Source: J Microbiol. 2026 Jan 31;64(1):e2510010. doi: 10.71150/jm.2510010 (PMC13576999; doi:10.71150/jm.2510010)
Supplement: Supplementary file 6 [file jm-2510010-Supplementary-Fig-S6.pdf]

Fig S6.

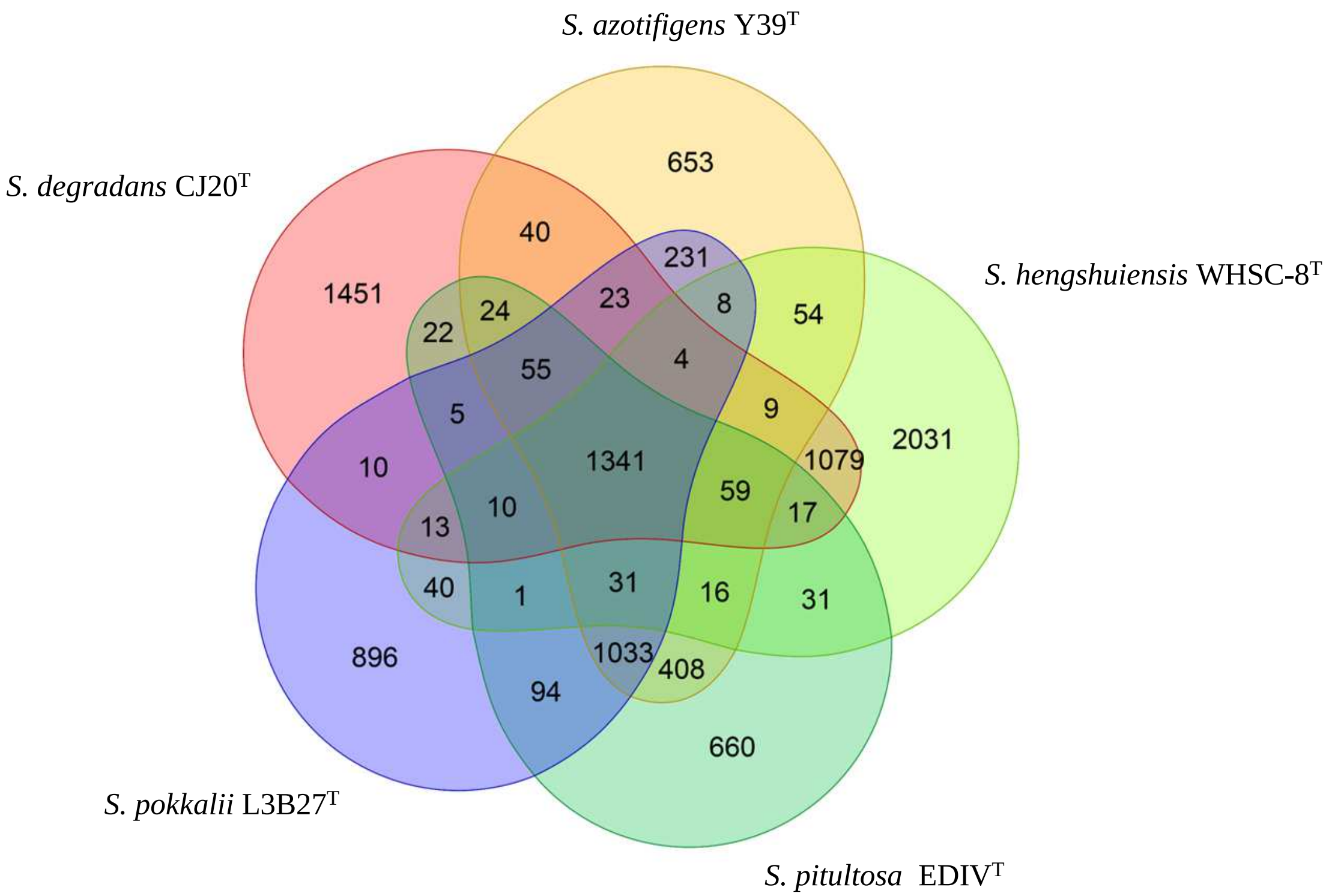
S. azotifigens Y39^T
S. degradans CJ20^T
S. hengshuiensis WHSC-8^T
S. pokkalii L3B27^T
S. pitultosa EDIV^T
653
40
1451
231
24
23
8
54
22
55
4
5
9
2031
10
1341
1079
59
13
10
17
40
1
31
16
31
896
1033
408
94
660

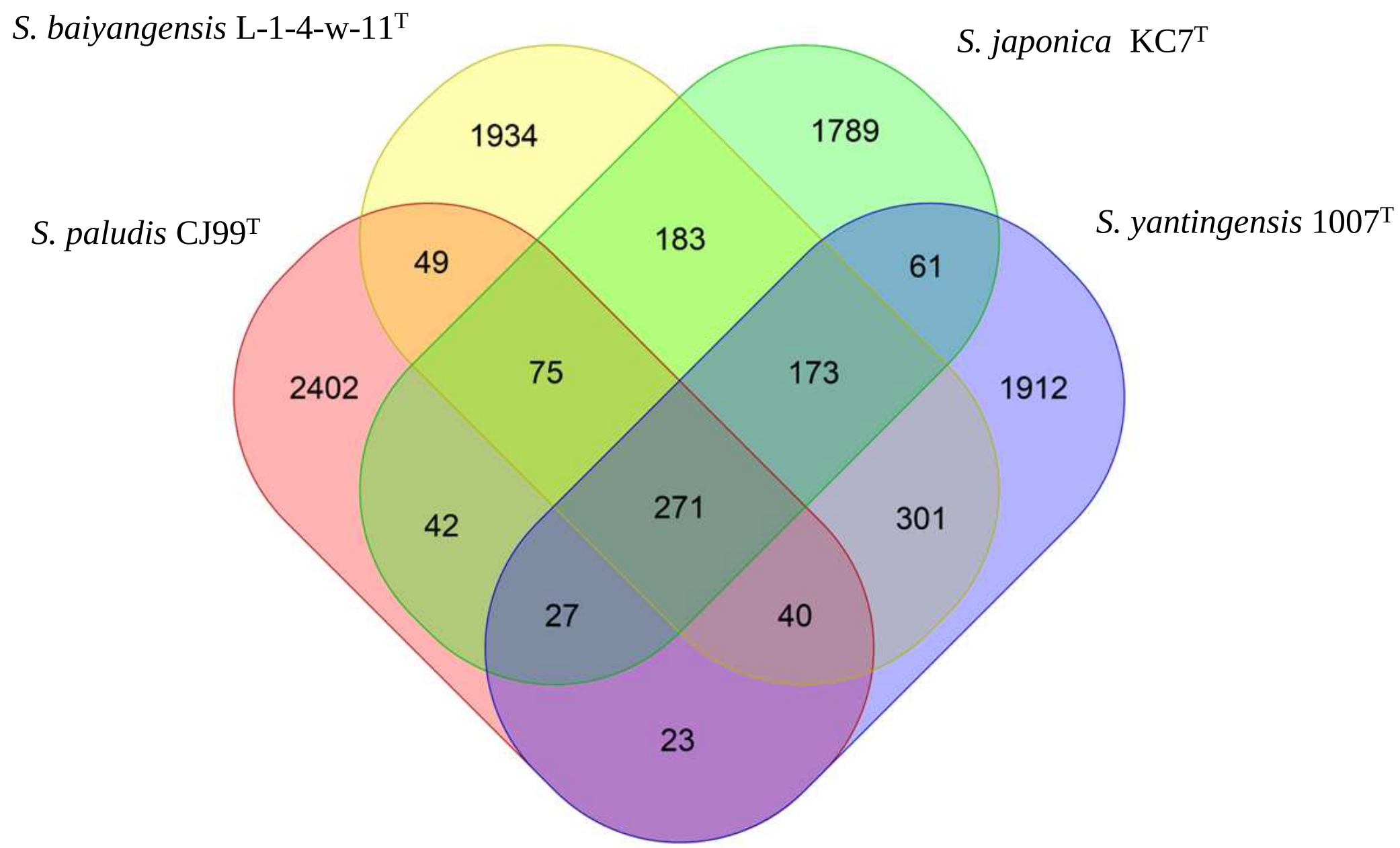
S. baiyangensis L-1-4-w-11^T
S. japonica KC7^T
S. paludis CJ99^T
S. yantingensis 1007^T
1934
1789
49
183
61
2402
75
173
1912
42
271
301
27
40
23
