# Supplementary material for: Sphingomonas degradans sp. nov. and Sphingomonas paludis sp. nov., isolated from the Han River and a wetland in South Korea
Source: J Microbiol. 2026 Jan 31;64(1):e2510010. doi: 10.71150/jm.2510010 (PMC13576999; doi:10.71150/jm.2510010)

Fig. S8.

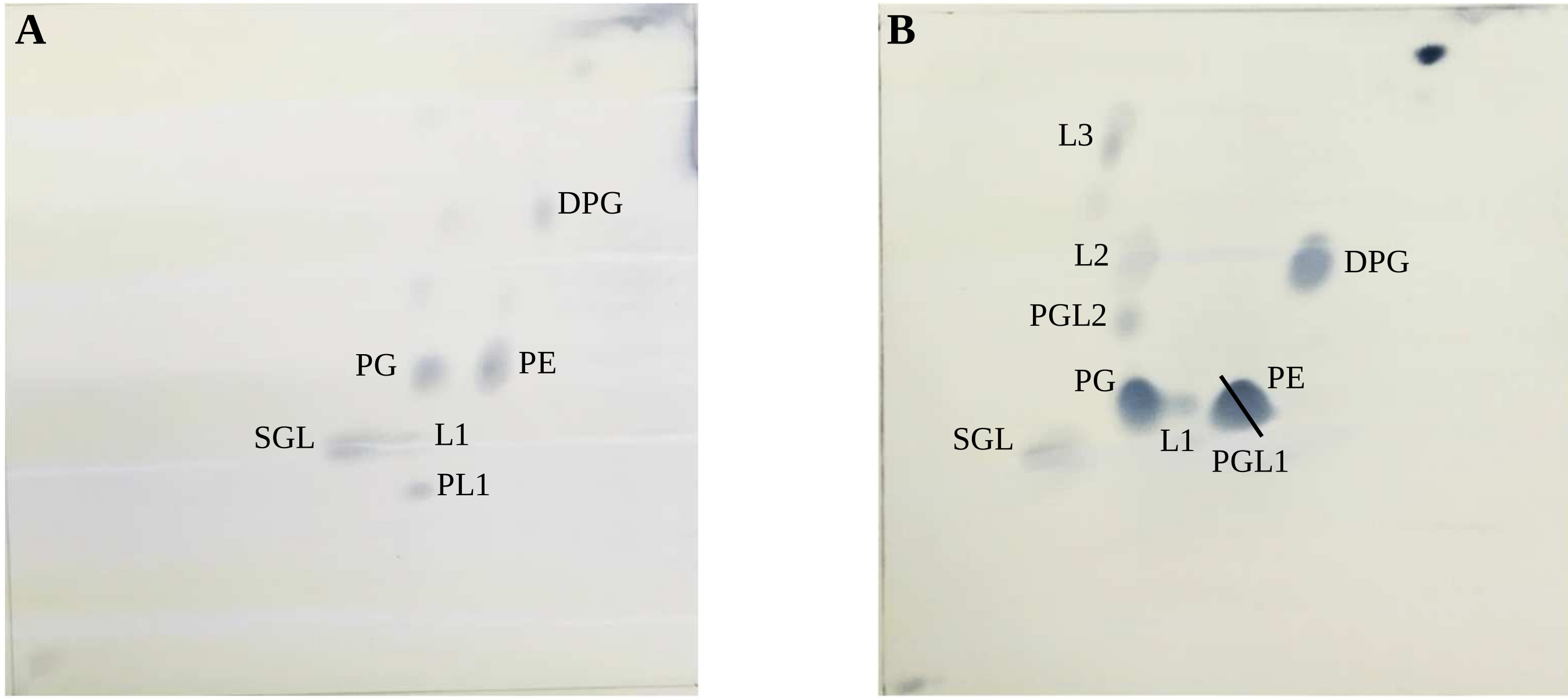

Supplement: Supplementary file 8 [file jm-2510010-Supplementary-Fig-S8.pdf]
